# Evaluation of a Novel System for RFID Intraoperative Cardiovascular Analytics

**DOI:** 10.1109/JTEHM.2022.3196832

**Published:** 2022-08-05

**Authors:** William Hendricks, Joshua Mecca, Maham Rahimi, Manuel R Rojo, Moritz C. Wyler Von Ballmoos, Ross G. McFall, Paul Haddad, Marton T. Berczeli, Kavya Sinha, Rebecca G. Barnes, Eric K. Peden, Alan B. Lumsden, Thomas E. MacGillivray, Stuart J. Corr

**Affiliations:** M&S Biotics Inc. Houston TX 77021 USA; Texas A&M Intercollegiate School of Engineering Medicine (ENMED) Houston TX 77030 USA; Department of Cardiovascular SurgeryHouston Methodist Hospital 23534 Houston TX 77030 USA

**Keywords:** Asset management, cardiothoracic surgery, vascular surgery, intraoperative analytics, radiofrequency identification, RFID tags

## Abstract

*Objective:* To evaluate a novel technology for real time tracking of RF-Identified (RFID) surgical tools (Biotic System), providing intraoperative data analytics during simulated cardiovascular procedures. Ineffective asset management in the Operating Room (OR) leads to inefficient utilization of resources and contributes to prolonged operative times and increased costs. Analysis of captured data can assist in quantifying instrument utilization, procedure flow, performance and prevention of retained instruments. Methods & *Results:* Five surgeons performed thirteen simulated surgical cases on three human cadavers. Procedures included (i) two abdominal aortic aneurysm (AAA) repairs, (ii) three carotid endarterectomies (CE), (iii) two femoropopliteal (fem-pop) bypasses, (iv) thoracic aortic aneurysm repair, (v) coronary artery bypass graft, (vi) aortic valve replacement, (vii) ascending aortic aneurysm repair, (viii) heart transplants, and (ix) mitral valve replacement. For each case an average of 139 surgical instruments were RFID-tagged and tracked intraoperatively. Data was captured and analyzed retrospectively. Of the 139 instruments tracked across each of the 13 cases, 55 instruments (39.5%) were actually used, demonstrating a high level of redundancy. For repeat cases (i.e. CE/AAA/fem-pop): (i) average instrument usage was 41 ± 3.6 (8.8% variation) for CE (n=3); (ii) average instrument usage was 69 ± 4.0 (5.8% variation) for AAA (n=2); and (iii) average instrument usage was 48 ± 2.5 (5.3% variation) for fem- pop (n=2). Results also showed a reduction in end-of-procedure instrument counting times of 58-87%. *Conclusions:* We report on a method for collecting intraoperative data analytics regarding instrument usage via RFID technology. This system will help refine instrument selection, quantitate instrument utilization and prevent inadvertent retention in a patient. This should help increase efficiency in packaging and sterilization and let surgeons make objective decisions in the composition of surgical trays. *Clinical and Translational Impact Statement*—Intraoperative analytics of surgical tools and associated equipment may ultimately lead to safer more efficient surgeries that increase patient outcomes while decreasing the cost of care.

## I. Introduction

Prolonged operative times are associated with significant patient morbidity and mortality, and multiple studies have quantified the degree to which prolonged operative time increases a patient’s risk of adverse complications, like infections. The likelihood of surgical site infections (SSIs), for example, increases with operative time (13%, 17%, and 37% for every 15 min, 30 min, and 60 min of surgery, respectively), and the risk of other complications increases by 14% every 30 min [1]. Another analysis of almost 10,000 plastic surgeries showed a rise in the odds of morbidity of over 25% per hour [2]. Much of the correlation between SSIs and delayed operative time is related to time-based factors such as prolonged microbial exposure, decreased efficacy of antibiotics, and increased chances for sterile technique violation [3], [4], while other complications can be a result of Operating Room (OR) traffic [5], surgical team fatigue, and extended anesthetic duration. A recent meta-analysis of thousands of surgical procedures concluded that decreasing the operative time has the potential to not only drastically improve patient outcomes, but also increase efficiency and result in cost-savings for a hospital [4].

Similarly, ineffective asset management in the OR leads to inefficient utilization of resources and may contribute to prolonged operative times. The current methods for determining which surgical tools are required in a specific case are severely outdated, interrupt workflow, and ultimately lead to longer procedures. Often, surgeons use preference cards to manually list their preferred items for a procedure, basing their needs on individual training rather than quantified usage best-practices [6]. Sadly, in many cases, the protocol for individuals to change a hospital’s standing order is so arduous and time-consuming that multiple procedures go by before teams have the equipment that they need [7]. The larger problem of inaccurately standardized sets becomes a factor because case-specific sets are created based on archaic and unconfirmed assumptions of usage from individual preference [8]. This ultimately leads to each custom set being compiled such that it may contain many technique-specific tools that surgeons will never touch and too few of those they need, leading to fewer than 20% of the tools in surgical sets being frequently (if ever) used [8], [9].

Once a surgical set arrives in the OR, hospitals rely on manual processes to keep track of surgical tools and compile surgical sets before, during, and after the procedure. Unfortunately, 12.5% of surgeries result in a counting discrepancy (i.e., incorrect count, item misplacement, or documentation error). In the cases with a retained surgical instrument (RSI), 88% occur with a reported accurate count [10]. Not only is this process antiquated and inaccurate, but it is needlessly time-consuming. Accounting for sponges alone consists of roughly 14% of the average operative time, not including the extra 20 minutes required to resolve a count discrepancy [11], [12]. For perspective, in a 180-minute prostatectomy without counting discrepancies, it can take nearly 25 minutes to account for 50–60 sponges [11]. Multiple studies have noted that if surgical teams were more familiar with the procedural equipment and its location thereof, such pre-operative planning could reduce the amount of time spent making critical decisions during operation and allow the anticipation of any additional equipment needed [1], [13]. Unfortunately, there is currently no way to collect the data to quantify pre-operative planning optimization, equipment usage, nor any of the countless metrics that define best practices in the OR.

### A. The Biotic System

M&S Biotics has developed an autonomous and cost-effective tracking system (Biotic System) for the OR that uses proprietary RFID technology to passively detect, count, track, and locate surgical items in real time. The true benefit of M&S’ solution is two-fold: (1) patented hardware leading to real-time tracking of equipment within the OR, and (2) proprietary software analytics of all procedural data-points, allowing for a never-before-seen quantifiable metric of surgical equipment usage. In the long term, the compounded data analytics will provide insight into surgical sets and surgical techniques, reducing the amount of redundant equipment and eliminating the processing costs associated with unused items. This represents the first system capable of passively collecting data related to instrument utilization and the first conduit for accessing never-before-quantified surgical data, enabling the possibility of correlating any technique or OR procedure with patient outcomes.

The use of RFID technology, specifically passive Ultra-High Frequency (UHF) which falls between 902–928 MHz, in an OR setting is difficult due to the nature of the items being tracked. Some of the issues that create these challenges for metal instruments in particular include tag detuning, re- radiation cancellation, and tag shadowing [15].

Tag detuning is a result of the power loss due to a mismatch between tag antenna and an integrated circuit (IC) originating from the impedance change of the tag antenna [14], [15]. Essentially, tags in a close relationship may absorb power from one another, and “de-tune” each other’s antenna thereby hampering the tag’s ability to receive signals from the interrogator. The introduction of metal surfaces impedes the originating RF wave and further contributes to potential errors from tag detuning [14], [15]. Additionally, the combination of the two electromagnetic signals (generated by both tag and reader) has the potential to negate the other’s signal at least partially in the form of tag re- radiation cancellation [14], [15]. This is where the re- radiated waves from the RF tag couple with the produced RF wave from the reader to combine in a manner that leads to interference [14], [15]. In certain instances, this combined signal interference is destructive and may prevent accurate implement detection or prevent tag detection altogether. Tag shadowing may also occur where the ability of tags closer to the excitation sources (RF reader) are disproportionately affected by the electromagnetic waves generated from the RF reader and capture more energy than tags further away [14], [15]. The tags at a farther distance from the excitatory impulses of the reader may be masked and read inefficiently. These tags are essentially “shadowed” by more immediate tags, and the tags closer to the reader’s impulses are received with greater clarity.

**FIGURE 1.**
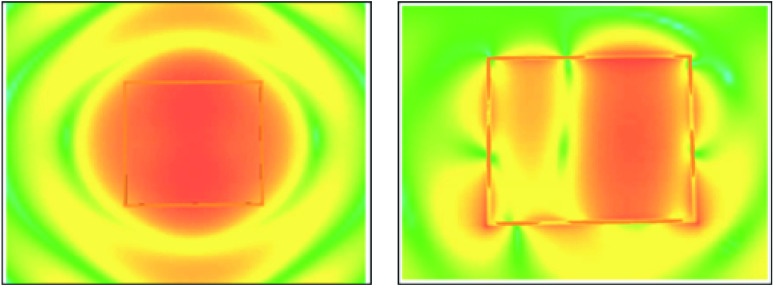
Beam propagation and RF spillover Left: Standard UHF RF field. Right: Dynamic RF field generated by M&S Biotics’ antenna element.

The ORLocate®System by Haldor Advanced Technologies, the Situate^™^ Detection System by Medtronic, and the system developed by CareTag are examples of technologies that leverage RFID in a surgical setting. Existing technology must recognize and account for all the limitations (e.g., tag detuning, re- radiation cancellation, and tag shadowing) in the field. One way to account for these limitations is to implement a manual work around, where the user must manually steer the reader-generated RF field in multiple directions so as to not create destructive interference on the back-scatter communication. For example, if a reader is producing a field in an omnidirectional fashion, the accuracy may deteriorate due to interference from tag shadowing and re- radiating cancellation. As waves progressing in all directions interrogate tags, the back-scatter communication causes destructive interference canceling out the RF signal originally sent. At the same time, tags that are further away from the wave capture inadequate energy for return transmissions due to tag shadowing and are either poorly activated or never activated. This results in inadequate or no re- communication with the reader. Figure 1 shows the beam propagation and RF spillover from a standard RFID antenna as well as that of the antennas within the Biotic System. The antenna’s within the Biotic System are designed to create a dynamic RF field that minimizes spillover and accounts for the likelihood of typical limitations such as detuning and shadowing.

## II. Objectives

**FIGURE 2.**
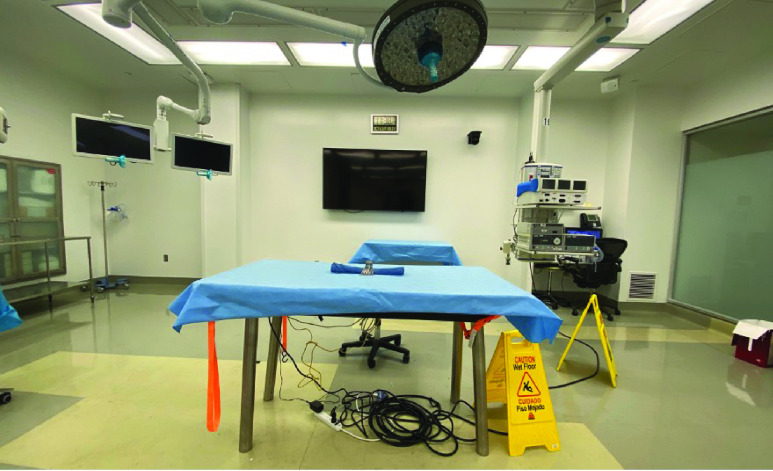
The M&S biotics back table within a simulated OR at the Methodist Institute for Technology, Innovation and Education (MITIE).

The purpose of this study was to evaluate the Biotic System Beta Prototype (Fig. 2) as a novel means of tracking RFID-enabled surgical tools during simulated surgical procedures as well as understanding potential data insights such a technology could quantify.

## III. Methodology

### A. Simulated Surgical Case Study Design

The protocol was created in part by researchers at M&S Biotics, Inc. as well as researchers at the Houston Methodist Institute for Technology, Innovation & Education (MITIE) in Houston, TX. The study enrolled cardiothoracic and vascular surgeons from the Houston Methodist Hospital Debakey Heart and Vascular Center to conduct simulated surgical cases on human cadavers. The simulated surgeries included acute vascular surgeries as well as invasive transplant procedures. The variety of cases conducted by surgeons of varying expertise allowed researchers to collect data points from procedures with different surgeon preference cards and instrumentation requirements. The cases were performed in the OR suites within MITIE, mimicking a real OR as closely as possible. Prior to each simulated procedure, each respective lead surgeon requested surgical instruments from standard hospital preference cards.

**FIGURE 3.**
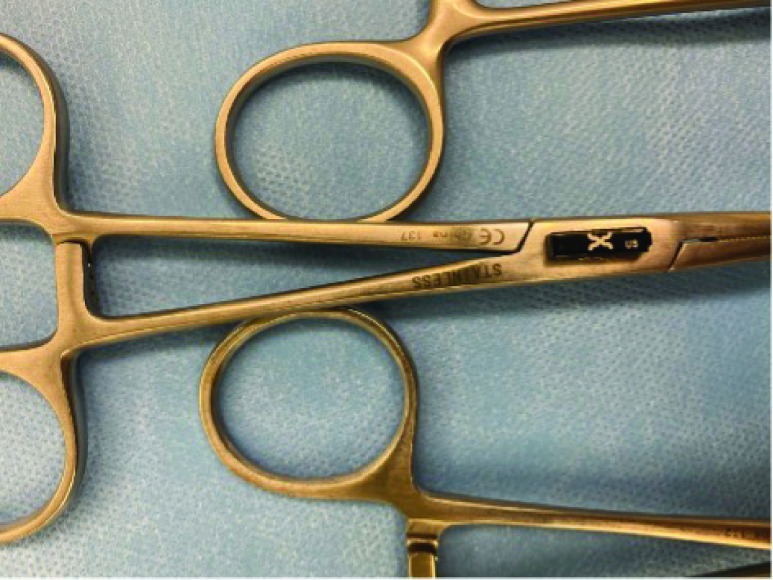
A Xerafy Dash XS tag affixed to a hemostat via super glue.

**FIGURE 4.**
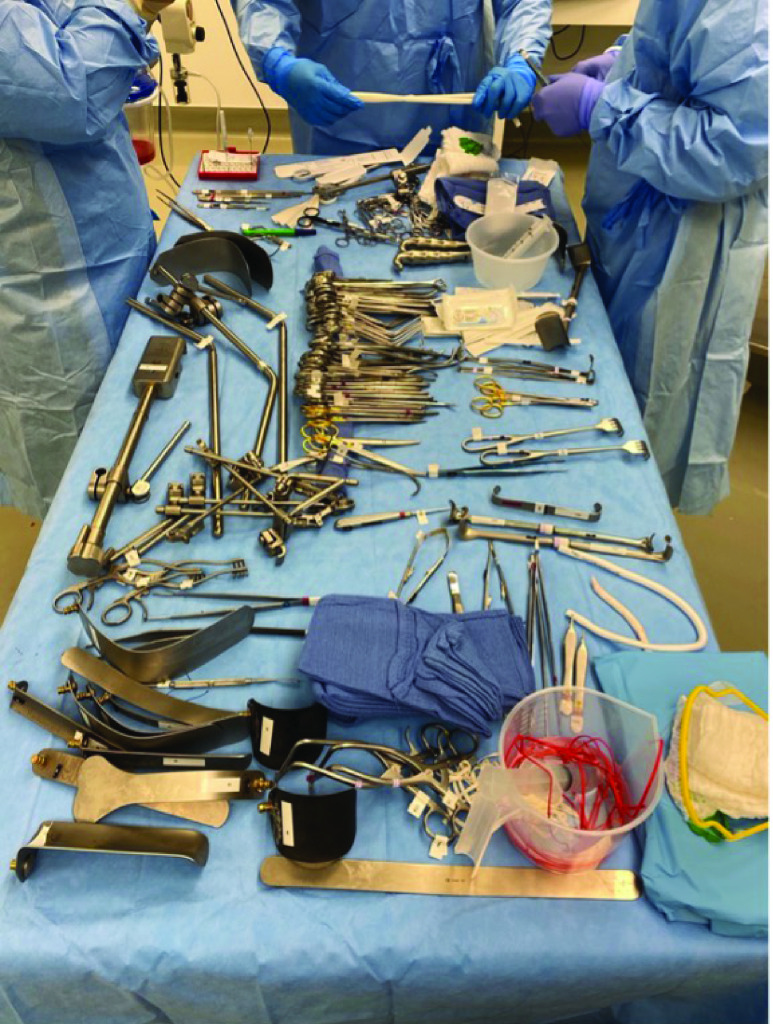
A surgical team standing around the back table, preparing for a procedure. Each metal item on the back table has been tagged with a Xerafy Dash XS RFID tag.

An experienced surgical skills specialist reviewed preference cards that requested high volumes of instruments and made available most of the items requested. Some redundant items requested were not included in the set due to resource constraints within the simulation facility as well as prior knowledge of what would not actually be utilized. Given that not all instruments requested on the preference card were made available, the surgeon was instructed to request any missing instruments he or she needed to perform the procedure. It is important to note that the operating surgeons did not request any additional items outside of the ones provided, despite being prompted to comment if any items they requested were not available and if they would be necessary to conduct the procedure. Each instrument requested was semi-permanently retrofitted with standard 902–928 MHz RFID tags designed to comply with FDA requirements for Unique Device Identification (Fig. 3) and laid out according to standard practice by an experienced surgical technologist (Fig. 4). The Biotic System was used in place of a standard back table and was controlled and monitored only by researchers from M&S Biotics, Inc. The surgical teams were instructed to conduct the simulated procedure as they would in a live case and the teams were not briefed on the Biotic System in order to prevent any bias during instrument utilization.

As each instrument was tagged with a unique RFID tag, researchers from M&S Biotics monitored instrument movement using the Biotic System interface. The System began interrogating tags at the beginning of the procedure, while all items were still on the back table, and ceased interrogating tags when all items returned to the back table at the conclusion of each procedure. To verify accuracy of instrument tracking, the surgical technologists assisting during the procedure were told to say out loud which items were being pulled from the back table during the procedure and which ones were returning. This data was manually recorded along with its associated timestamp.

### B. Counting Study Design

In order to isolate the process by which surgical technologists and surgical nurses count in and prepare surgical instruments prior to a surgical case, 4 teams of technologists and nurses were separately asked to count in various instrument sets. The counting procedures were conducted at the Operating Suites within MITIE at Houston Methodist Hospital. Each team was given 3 sets of surgical items, with corresponding count sheets, and was asked to count them in as they would prior to an actual surgical procedure. The teams were instructed to use the Biotic System in lieu of a typical table, laying out each instrument as they normally would before a case after pulling them from the instrument set.

The teams were instructed to count one set at a time. The first set contained 75 instruments, the second contained 98 and the third contained 125. For sets one and three (75 and 125 instruments, respectively), the count sheet provided with the instrument set was accurate. For set two (98 instruments), the count sheet incorrectly listed 100 items. This error was introduced to mimic a real-life scenario that happens frequently to surgical teams counting in instruments for certain cases. In doing so, the researchers were able to better understand how surgical teams handle count discrepancies during a procedure and show the Biotic System’s ability to accurately display all available instruments regardless of what instruments should or should not be present according to the count sheet. The teams were instructed to behave as they normally would when encountering an incorrect count or count sheet. The total time to successfully reconcile all the items in each set with each count sheet was recorded. To compare the Biotic System’s ability to interrogate and count items, the system began interrogating items when the teams began counting and ceased when the teams stopped counting. The total time for the Biotic System to successfully reconcile all the items in each set was recorded.

### C. Surgical and Count Procedure Design

For each surgical procedure, the total number of instruments requested by each surgeon was recorded. The total number of instruments requested per case was the sum of the total instrument counts from each instrument count sheet. The number of instruments actually used during each case was recorded and compared with the total number of instruments requested. For different surgeons who conducted the same cases, their instrument utilization was also compared.

For each counting study, the duration of time the Biotic System took to count all RFID-enabled items was compared with the duration of time each participating team took to manually count each item.

## IV. Results

### A. Instrument Utilization

**FIGURE 5.**
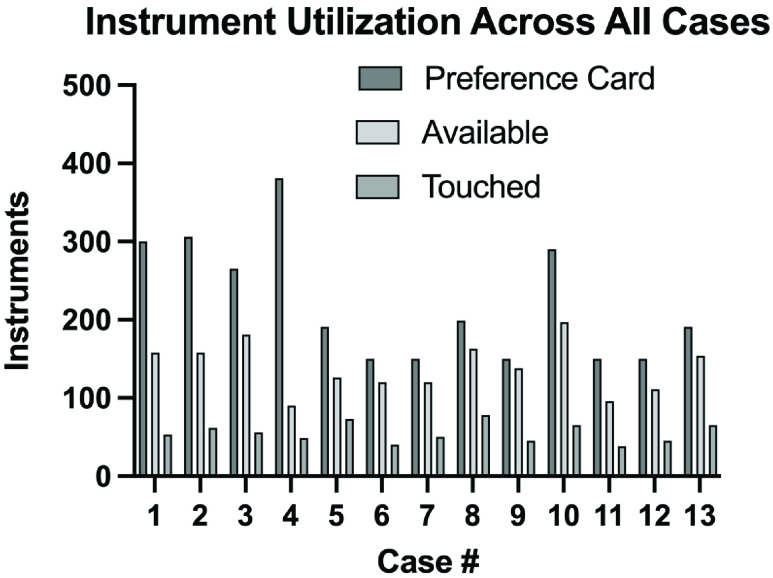
The number of metal instruments requested on the preference card for each case (Preference Card), the number of items made available for each case by an experience surgical skills specialist prior to the procedure (Available), and the number of instruments actually utilized during the procedure (Touched).

**FIGURE 6.**
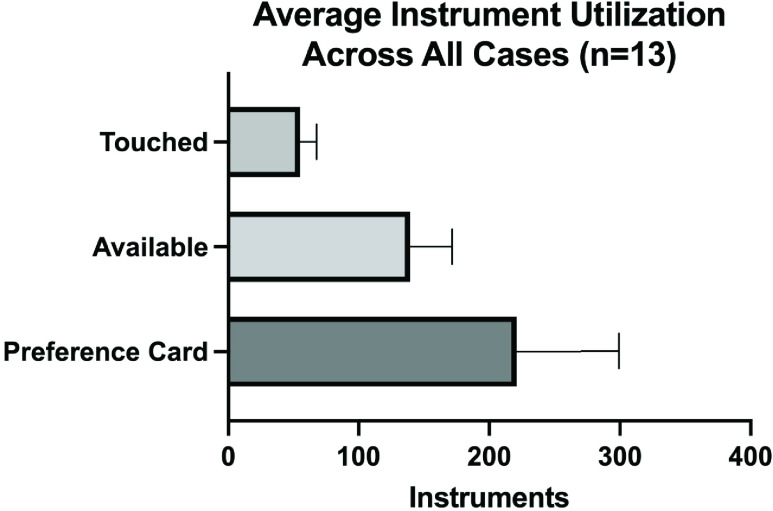
The average number of metal instruments requested on preference cards (Preference Card), the average number of items made available (Available), and the average number of items actually used by the surgical team across all 13 cases (Touched).

Five different surgeons performed a total of thirteen simulated surgical cases on three separate human cadavers. The cases performed included the insertion of a coronary artery bypass graft (CABG), an aortic valve replacement (AVR), an ascending aortic aneurysm repair, a thoracic aortic aneurysm repair, a total heart transplant, two abdominal aortic aneurysm repairs (AAA), three carotid endarterectomies (CE), two femoropopliteal (Fem-Pop) bypass surgeries, and a mitral valve replacement. Raw data from these 13 studies can be found in Table 1 with a corresponding graphical illustration shown in Fig. 5. Average data is depicted in Fig. 6. Across all cases, an average of 221 ± 78.1 instruments were requested via the surgical preference cards (Fig. 6). Of those 221 ± 78.1, 63.07% of the instruments were prepared for the case. On average, 139.4 ± 32.4 instruments were made available and prepared for each case.

**TABLE 1:** The Instruments Utilized During Each of the 13 Cases Observed. The Number of Instruments Listed on the Preference Cards, the Instruments Made Available for the Surgical Procedure, and the Number of Instruments Actually Touched or Used During the Case are Listed Above. The Percentages of the Instruments Requested on the Preference Cards That Were Touched are Similarly Listed

Instrument Volume and Utilization Across All Cases				
Case	Instruments on Preference Card	Instruments Available	Instruments Touched	
Coronary Artery Bypass Graft (CABG)	300	158	53	17.67%
Aortic Valve Replacement (AVR)	306	158	62	20.26%
Ascending Aortic Aneurysm Repair (AAR)	265	181	56	21.13%
Total Heart Transplant	381	90	49	12.86%
Abdominal Aortic Aneurysm Repair (AAA)	191	126	73	38.22%
Carotid Endarterectomy (CE)	150	120	40	26.67%
Femoropopliteal (Fem-Pop) Bypass	150	120	50	33.33%
Thoracic Aortic Aneurism Repair	199	163	78	39.20%
Carotid Endarterectomy (CE)	150	138	45	30.00%
Mitral Valve Replacement	290	197	65	22.41%
Carotid Endarterectomy (CE)	150	96	38	25.33%
Femoropopliteal (Fem-Pop) Bypass	150	111	45	30.00%
Abdominal Aortic Aneurysm Repair (AAA)	191	154	65	34.03%
AVERAGE	221 ± 78.1	139.4 ± 21.4	55.3 ± 12.5	25.03%

Of the total instruments requested on the preference cards for each case (221 ± 78.1), only an average of 25.03% of the instruments were actually touched or used by the surgical team during the case. Even after the significant 63.07% reduction in the set sizes by an experienced surgical skills specialist, only 39.68% of those 139.4 ± 32.4 available instruments were touched or used by the surgical team.

Three cases were repeated by different surgeons, the CEs, the Fem-Pop bypasses, and the AAA repairs (Table 2). See Table 2 for the volume of instruments requested for each case and the number of instruments actually utilized for each case. An average of 41 ± 3.6 instruments were used across the three carotid endarterectomy cases, representing 27.33% of the requested instruments. Those conducting the two femoropopliteal bypass cases utilized approximately 48 ± 2.5 items for each case, representing 32.00% of the requested instruments, and those conducting the two AAA repairs utilized approximately 69 ± 4.0 instruments for each case, representing 36.13% of the requested items.

**TABLE 2:** This Table Lists the Instrument Volume Requested for Each Case (Instruments on Preference Card) and the Total Instruments Actually Utilized. The Instruments Utilized are Listed as the Number for Each Case as Well as the Percentage of the Instruments Requested on the Preference Card. The Average for Each Case is Shown

Instrument Utilization in Repeat Cases			
Case	Instruments on Preference Card	Instruments Touched (#)	Instruments Touched %
Carotid Endarterectomy (CE)	150	40	26.67%
		45	30.00%
		38	25.33%
	AVERAGE	41 ± 3.6	27.33%
Femoropopliteal (Fem-Pop) Bypass	150	50	33.33%
		45	30.00%
	AVERAGE	48 ± 2.5	32.00%
AAA Repair	191	65	34.03%
	AVERAGE	69 ± 4.0	36.13%

### B. Counting Study

A total of four teams of surgical technologists and circulating nurses participated in the counting study, each counting three sets of instruments. See Table 3 for the mean manual and automated count times in each trial. On average, the Biotic System was able to read 76.5s, 195.9s, and 191.3s faster than the manual teams for each of the three trials respectively (Fig. 8). As illustrated in Table 3, use of the automated counting system led to a decrease in counting time relative to manual counts by an average 58.85%, 74.36%, and 87.32% for each trial, respectively. Due to an error in tag fixation (e.g., tag loosely affixed to instrument, tag removed from instrument, etc.) the Biotic System only captured all 125 instruments in set three during two of the four trials. Instead, the Biotic System was only able to capture 124 out of 125 items in two of the four tests. Table 3 shows that for the two tests where the system successfully captured all 125, the mean automated count time was 75 ± 52.3 seconds. The system was able to capture 124 out of 125 instruments in all four tests and the mean automated count time was 28.0 ± 8.1.

**TABLE 3:** This Table Lists the Times it Took Each Team to Count Each Set, and the Corresponding Times for the Biotic System to Count the Same Set. The Corresponding Percent Decrease in Counting Time From the Manual Method to the Automated Method Using the Biotic System is Shown

Manual vs Automated Instrument Counting Time (n=4)			
	Mean Count Manual (s)	Mean Count Biotic System (s)	Mean Decrease in Count Time
Trial 1 (75/75)	130 ± 49.7	53.5 ± 77.7	58.85%
Trial 1 (74/75)	–	18.3 ± 12.7 (n=3)	–
Trial 2 (98/98)	263.3 ± 71.1	67.5 ± 46.8	74.36%
Trial 2 (97/98)	–	42 ± 34.8	–
Trial 3 (125/125)	266.3 ± 81.9	75 ± 52.3 (n=2)	87.32% (n=2)
Trial 3 (124/125)	–	28.0 ± 8.1	–

**FIGURE 7.**
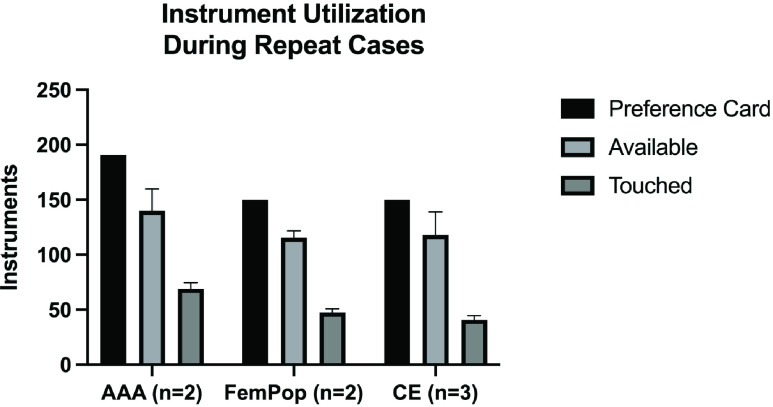
Average instrument utilization for the AAA Repairs (n=2), Fem-Pop Bypasses (n=2), and CEs (n=3) conducted.

**FIGURE 8.**
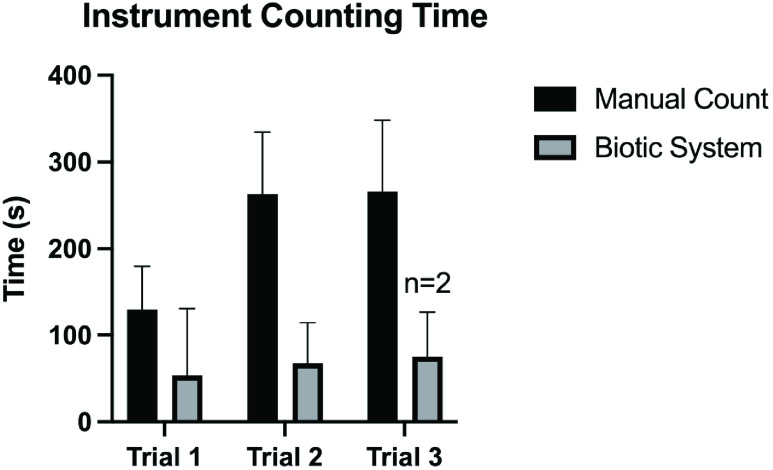
The average time in seconds it took the surgical teams to manually count the instruments from each trial, and the average time in seconds it took the biotic system to count the instruments in each trial. Please note that the sample size for the Biotic System in Trial 3 was n=2.

## V. Discussions

### A. Overall Instrument Utilization

There are many reasons why surgical teams would request more instruments than necessary for each case. Much of the surgical staff cited the need for tools to conduct emergency procedures when asked why more instruments may be requested than actually utilized. Overall, many more instruments were requested for each case than were necessary. This was evident by an average 25.03% utilization rate of the 221 ± 78.1 items requested. In addition to the number of items requested on the preference card and the number of items utilized during each case, an important metric included in the data was the number of surgical tools made available for each procedure. Prior to each case, a highly experienced surgical skills specialist reviewed the intended procedure and the surgeon leading the case. Given her understanding of each surgeon’s preference, she made available only those items she felt would be frequently used, attempting to not prepare redundant instruments that would only serve to clutter the back table. Intuitive reduction of redundant surgical sets is already a present phenomenon in the minds of many experienced surgical teams, but the data does not yet exist that would allow them to make these set optimizations without fear of needing an instrument that was removed. That said, only 39.68% (55.3 ± 12.5 instruments) of the instruments pulled by the experience surgical skills specialist was utilized compared to the 139.4 ± 32.4 available. This data shows that roughly a quarter of the instruments on a preference card were actually used during each case and less than half of the instruments that were estimated to be utilized were actually used. It is also important to note that while not all of the requested items were provided for each case, no surgeon nor surgical team requested an additional item that was not provided.

Of course, procedures on cadavers are unlikely to have unintended complications that require additional procedures to mitigate any unintended bleeding disasters, for example, but this low utilization rate points towards the ability to optimize surgical sets in the future. The processing protocol for each surgical instrument is time intensive and costly, and optimization could save costs, reduce pre-operative preparation time, reduce overall operative time and reduce clutter in the OR. Significant surgical set optimization has yet to become a widely adopted practice because no one wants to cut instruments from a set that may be required even in the rarest of emergency cases. That said, an innovation that would allow for the quantification of instrument utilization while in the OR for specific cases over time would provide one aspect of data from which administrators and clinicians could make educated set optimization decisions.

Certain instruments that are never used over a long period of time, and were perhaps only included due to archaic procedures or techniques that were once considered standard but are no longer frequently utilized, may be removed completely. Instruments that are used rarely, but still often enough that they should not be completely eliminated from the set, could be designated as rare-use items and processed separately. Using this one example of alternative processing, instruments used frequently may be processed and prepped for each case, but those only rarely used may be kept in separate sterilized packages that may or may not be opened during each case. If these emergency sets were only opened when needed, they would not have to be unnecessarily processed at the end of each case. Amongst other benefits, this may extend the lifetime of each instrument set.

### B. Manual Versus Automated Counting Times

Instrument set reconciliation, that is counting all the instruments being used during a procedure and verifying the instruments with the corresponding count sheets, is a process that occurs many times before, during and after a surgical procedure. All members of the surgical staff are involved in at least some capacity. In essence, the purpose is to ensure the team has the required instrumentation for the case and that nothing is left behind in the patient once the case has concluded. By automating this counting process and significantly decreasing the time required to count in each instrument set, the surgical staff can focus their attention on other necessary aspects of the procedure as well as reduce the amount of time spent counting. Reducing the amount of unnecessary operative time in any procedure is welcomed and imperative because each minute reduced reduces the risk of complications.

Over time, as sets become larger and more inundated with instrumentation and potential errors in the count sheets, they became more difficult for the surgical team to count. While counting Set two, various members of the surgical team accounted for the programmed discrepancy and made a note on the count sheet that certain items were not on the table that were listed on the count sheet and manually wrote the correct number of total instruments. A few members, however, did not catch this error at first and were prompted to restart the counting process if they were unsure if all instruments were present.

For the purposes of this experiment, the RFID tags were semi-permanently affixed to each instrument. Unfortunately, the method for fixation was not as secure as a standard FDA-cleared epoxy used to attach RFID tags to instruments. If the tag became loose due to movement or adhesive degradation, the strength of the signal propagated by the tag required to be read quickly by the system was not always guaranteed. In short, permanent attachment of the RFID tag to the instrument would undoubtedly reduce error and improve performance of the system.

**FIGURE 9.**
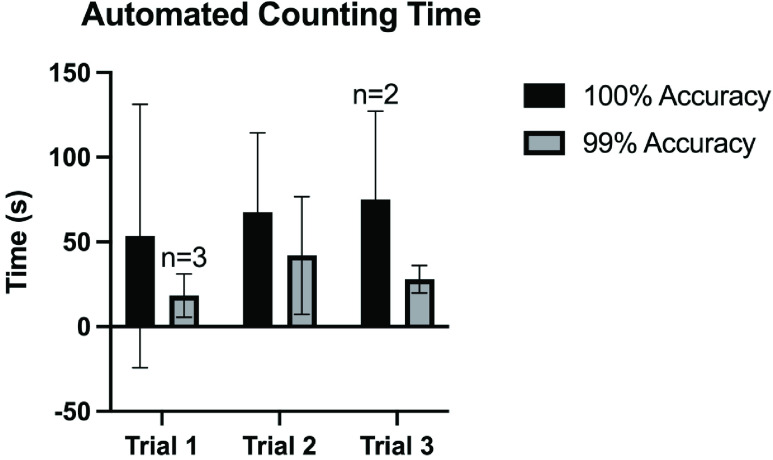
The average time it took the biotic system to count in all of the instruments in each of the three trials as well as the amount of time taken to count all but one of the instruments in each of the three trials.

**FIGURE 10.**
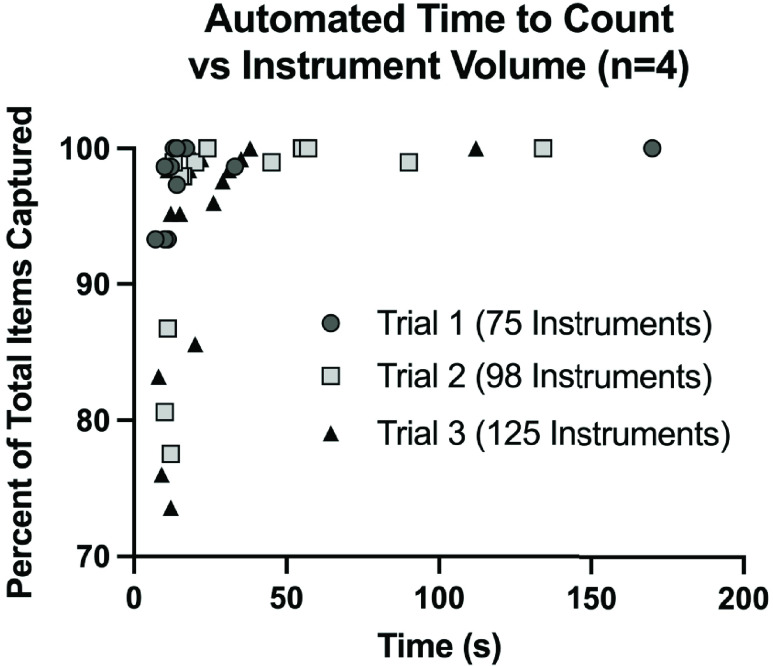
Each of the data points collected from the biotic system while counting during the counting study for all four tests during all three trials. Each data point is plotted as a function of the percent of the total instruments read versus time. These measurements were taken continuously while counting in each total set.

When monitoring the Biotic System’s user interface to keep note of count accuracy and timing, an interesting phenomenon was observed. As hypothesized, the larger instrument sets were observed to take longer to count. That said, there was a significant difference observed between the time it took to count all of the instruments versus all but one of the instruments in each data set. The Biotic System was able to reach all instruments during Trial 1 while being counted in by Teams 1, 3 and 4 in 17 seconds, 13 seconds, and 14 seconds, respectively. The Biotic System read all 75 instruments during Trial 1 in 170 seconds while the instruments were being counted by Team 2, but was able to read 74 out of 75 seconds in 33 seconds. This data shows that the Biotic System was searching for the last unread tag during this trial for 137 seconds, or just over 2 minutes. Similarly, Table 3 lists the Biotic System took an average of 67.5 ± 46.8 seconds to count all 98 instruments in Trial 2, but an average of 42 ± 34.8 seconds to count 97/98 instruments. The longest observed gap in counting time occurred between counting 99% of the instruments, regardless of volume, and counting 100% (Fig. 9). This observation can be the result of many variables, including shadowing and detuning due to the spatial orientation of the tagged instruments in relation to the other items. That said, Figure 10 shows that the system was able to capture the majority of the instruments within the field, independent of the volume of instruments present. This observation suggests that the problem does not lie within the Biotic System’s ability to fully saturate all tagged instruments within range, but difficulty capturing the elusive last item may be a result of some aspect of the current prototype’s analytic capability.

While an average 58.85%, 74.36%, and 87.32% reduction in instrument counting time is significant, a full surgical set with RFID tags fitted using a durable FDA-cleared epoxy may yield even more stable and accurate results. Further study into the accuracy of the system controlling for variables such as the proximity of RFID tagged items to one another and the orientation of the tags and instruments in 3D space is warranted.

### C. Adopting RFID Technology: Additional Considerations

Many believe that RFID technology will represent the future of healthcare asset management, but some have also expressed concerns on the practical application of such technology in a surgical setting. RFID technologies traditionally track the RFID tags themselves, and the presence or location of certain items is assumed due to the presence of the attached tag. In the instance that the tag becomes detached from such an item, that item can no longer be tracked. One goal of digital asset management in surgery is to prevent retained surgical items, but this is not possible if the item being tracked loses its connection with the RFID tag. Similarly, if the signal from the tag is no longer read for any reason, for example due to tag shadowing, the presence or location of an item may be incorrectly assumed. As RFID tracking technologies in healthcare have gained traction, many businesses and institutions have developed validated tag fixation processes that minimize the risk of a lost tag and missed instrument. These processes for attachment often contain a fixation medium (e.g. epoxy) that can be incorporated into the typical tag sterilization and processing. The tags themselves, such as the Xerafy Dash-XS tags used in this study, are autoclavable for many cycles and, in some instances, may have a longer use life than the instrument they are attached to.

From a safety point of view, new technologies with medical applications must adhere to certain standards and regulatory requirements as stipulated by the FDA and other international regulatory agencies. To ensure new electrical equipment does not create additional risks for patients or providers, many technologies similar to the Biotic System may require adherence and consideration of standards such as IEC 60601, which outlines safety and essential performance characteristics for medical electrical equipment. This is completed in order to mitigate risks such as interference with other medical technologies. Currently, the accepted UHF RFID bandwidth allowed in certain medical settings falls between 902–928 MHz.

## VI. Conclusion

This study is the first to report on a novel method for collecting intraoperative analytics regarding metal instrument usage via RFID technology. Until now, RFID technology in the OR has been widely regarded as a means of detecting retained surgical items at the conclusion of a procedure. The novel system for intraoperative data collection via RFID technology, the Biotic System, displays a clear ability to passively collect practical and applicable information during a surgical procedure. Similarly, it showcases a distinct ability to account for items in the OR much faster than current manual methods. It would be worth exploring the ability of a second generation of the device to monitor permanently affixed RFID-enabled surgical instruments during a live clinical setting, and studying any data collected from a longitudinal clinical study.

## References

[1] H. Cheng, B. P.-H. Chen, I. M. Soleas, N. C. Ferko, C. G. Cameron, and P. Hinoul, “Prolonged operative duration increases risk of surgical site infections: A systematic review,” Surgical Infections, vol. 18, no. 6, pp. 722–735, Aug. 2017. 28832271 10.1089/sur.2017.089 PMC5685201

[2] K. L. Hardy, “The impact of operative time on complications after plastic surgery: A multivariate regression analysis of 1753 cases,” Aesthetic Surgery J., vol. 34, no. 4, pp. 614–622, May 2014. 10.1177/1090820X14528503 24696297

[3] G.-Q. Li, F.-F. Guo, Y. Ou, G.-W. Dong, and W. Zhou, “Epidemiology and outcomes of surgical site infections following orthopedic surgery,” Amer. J. Infection Control, vol. 41, no. 12, pp. 1268–1271, Dec. 2013. 10.1016/j.ajic.2013.03.305 23890741

[4] H. Cheng, “Prolonged operative duration is associated with complications: A systematic review and meta-analysis,” J. Surgical Res., vol. 229, pp. 134–144, Sep. 2018. 10.1016/j.jss.2018.03.022 29936980

[5] G. Alizo, A. Onayemi, J. D. Sciarretta, and J. M. Davis, “Operating room foot traffic: A risk factor for surgical site infections,” Surgical Infections, vol. 20, no. 2, pp. 146–150, Feb. 2019. 30648925 10.1089/sur.2018.248

[6] (2018). Medtronic I. Engaging Surgeons to Enhance Care Delivery and Reduce Costs [Internet]. Accessed: Jun. 14, 2021. [Online]. Available: https://www.medtronic.com/au-en/transforming-healthcare/aligning-value/perspective/case-studies/ihs-surgeon-preference-cards.html

[7] L. Lingard, “Communication failures in the operating room: An observational classification of recurrent types and effects,” Qual. Saf. Health Care, vol. 13, no. 5, pp. 330–334, Oct. 2004. 15465935 10.1136/qshc.2003.008425 PMC1743897

[8] E. W. Stockert and A. Langerman, “Assessing the magnitude and costs of intraoperative inefficiencies attributable to surgical instrument trays,” J. Amer. College Surgeons, vol. 219, no. 4, pp. 646–655, Oct. 2014. 10.1016/j.jamcollsurg.2014.06.019 25154669

[9] R. S. Young and D. J. O’Regan, “Cardiac surgical theatre traffic: Time for traffic calming measures?” Interact. CardioVascular Thoracic Surgery, vol. 10, no. 4, pp. 526–529, Apr. 2010. 10.1510/icvts.2009.227116 20100706

[10] V. M. Steelman, “Sensitivity of detection of radiofrequency surgical sponges: A prospective, cross-over study,” Amer. J. Surgery, vol. 201, no. 2, pp. 233–237, Feb. 2011. 10.1016/j.amjsurg.2010.05.001 21266216

[11] C. K. Christian, “A prospective study of patient safety in the operating room,” Surgery, vol. 139, no. 2, pp. 159–173, Feb. 2006. 16455323 10.1016/j.surg.2005.07.037

[12] K. TKolb, T. Day, and W. McCall, “Accuracy of blood loss determination by health care professionals,” CRNA, vol. 10, no. 4, pp. 170–173, Nov. 1999. 10723295

[13] C. A. Willis-Owen, A. Konyves, and D. K. Martin, “Factors affecting the incidence of infection in hip and knee replacement,” J. Bone Joint Surgery. Brit. Volume, vol. 92-B, no. 8, pp. 1128–1133, Aug. 2010. 10.1302/0301-620X.92B8.24333 20675759

[14] Q. Zhang, M. Crisp, I. H. White, and R. V. Penty, “Power margin reduction in linear passive UHF RFID tag arrays,” in Proc. IEEE RFID Technol. Appl. Conf. (RFID-TA), Sep. 2014, pp. 11–306.

[15] Q. Zhang, M. J. Crisp, R. V. Penty, and I. H. White, “Reduction of proximity effects on UHF passive RFID systems by using tags with polarization diversity,” IEEE Trans. Antennas Propag., vol. 63, no. 5, pp. 2264–2271, May 2015.

